# Self-Consolidation Mechanism of Nanostructured Ti_5_Si_3_ Compact Induced by Electrical Discharge

**DOI:** 10.1155/2015/815084

**Published:** 2015-03-25

**Authors:** W. H. Lee, Y. W. Cheon, Y. H. Jo, J. G. Seong, Y. J. Jo, Y. H. Kim, M. S. Noh, H. G. Jeong, C. J. Van Tyne, S. Y. Chang

**Affiliations:** ^1^Faculty of Nanotechnology and Advanced Materials Engineering, Sejong University, Seoul 143-747, Republic of Korea; ^2^Department of Dental Laboratory Technology, Wonkwang Health Science University, Iksan 570-750, Republic of Korea; ^3^Center for SCINOVATOR, Posung High School, Seoul 138-829, Republic of Korea; ^4^Department of Metallurgical and Materials Engineering, Colorado School of Mines, Golden, CO 80401, USA; ^5^Department of Materials Engineering, Korea Aerospace University, Goyang-si 412-791, Republic of Korea

## Abstract

Electrical discharge using a capacitance of 450 *μ*F at 7.0 and 8.0 kJ input energies was applied to mechanical alloyed Ti_5_Si_3_ powder without applying any external pressure. A solid bulk of nanostructured Ti_5_Si_3_ with no compositional deviation was obtained in times as short as 159 *μ*sec by the discharge. During an electrical discharge, the heat generated is the required parameter possibly to melt the Ti_5_Si_3_ particles and the pinch force can pressurize the melted powder without allowing the formation of pores. Followed rapid cooling preserved the nanostructure of consolidated Ti_5_Si_3_ compact. Three stepped processes during an electrical discharge for the formation of nanostructured Ti_5_Si_3_ compact are proposed: (a) a physical breakdown of the surface oxide of Ti_5_Si_3_ powder particles, (b) melting and condensation of Ti_5_Si_3_ powder by the heat and pinch pressure, respectively, and (c) rapid cooling for the preservation of nanostructure. Complete conversion yielding a single phase Ti_5_Si_3_ is primarily dominated by the solid-liquid mechanism.

## 1. Introduction

Syntheses of intermetallic compounds with high melting points via mechanical alloying have been attempted in numerous studies [1, 2]. In general, combustion reactions have been initiated by ball milling in a variety of highly exothermic reaction mixtures. The formation of intermetallics from their elemental components accelerates during ball milling to become a self-sustaining high temperature reaction [3, 4]. Among intermetallic compounds, Ti_5_Si_3_ has attracted more interest recently because a number of their properties have potential in materials applications. Characteristics which make them promising high temperature structural materials include low temperature toughness, high temperature strength and creep resistance, oxidation resistance, and relatively low density [5, 6]. There are various ways to improve the fracture toughness of Ti_5_Si_3_, such as reduction of grain size and alloying with other elements [7–9].

Conventionally, a solid bulk typed Ti_5_Si_3_ can be synthesized by reacting mixed stochiometric powders of Ti and Si at higher temperature or arc melting of Ti and Si pieces [10, 11]. In spite of their research significance, in recent years there have been relatively few studies on the consolidation of Ti_5_Si_3_ in the form of powder. The usual sequence in powder metallurgy operations is to compact a metal powder in a die at room temperature and subsequently sinter it at elevated temperatures. Not only are high pressure, high temperature, and long times required, but in the case of reactive materials, such as Ti and its alloys, an inert atmosphere is also inevitably required. The high temperatures involved in these processes, however, result in detrimental changes in the microstructure and mechanical properties.

Lee and coworkers reported that Ti, Ti-6Al-4V, and Ti_5_Si_3_ powders can be successfully consolidated into a solid bulk type without detrimental changes in the microstructure and mechanical properties by using an electrical discharge technique [12–15]. However, the formation of nanostructured Ti_5_Si_3_ compact by the discharge has not been reported.

This paper thus analyzes the electrical discharge characteristics in terms of input energy, capacitance, and discharge time. It also systematically describes means by which the electrical discharge consolidates the mechanical alloyed Ti_5_Si_3_ powder particles to produce a solid compact with a nanostructure.

## 2. Materials and Methods

Elemental Ti and Si powders were mechanically alloyed (MAed) for 30 minutes at a fixed rpm of 1200 in an Ar atmosphere using a high speed Spex 8000D mixer/mill (SPEX Industries, Inc.) and a cylindrical partially stabilized zirconia (PSZ) vial (60 mm i.d. and 87 mm long) with high Cr hardened steel balls (10.0 and 4.7 mm in diameter). The charged atomic ratio of the reactants corresponded to the reaction stoichiometry (Ti-37.5 at.% Si). The purity of the powders was better than 99.95%. The mass of the powder charge was 10 g and the mass ratio of ball to powder was 5 : 1.

MAed powder without any surface treatment was mounted on the spectrometer probe tip by means of double-sided adhesive tape and examined by XPS (X-ray photoelectron microscopy) for any possible surface modification. Under the current conditions employed, the full width at half maximum (FWHM) of the Ag 3d_5/2_ peak was 1.1 eV, and the binding energy difference between Ag 3d_5/2_ and Ag 3d_3/2_ was 6.0 eV. When the Ag 3d_5/2_ peak was used as the reference peak, the binding energy of the C1s peak of adventitious carbon on the standard silver surface was 285 eV. All binding energies were referenced to the C1s peak to correct for sample charging.

**Figure 1:**
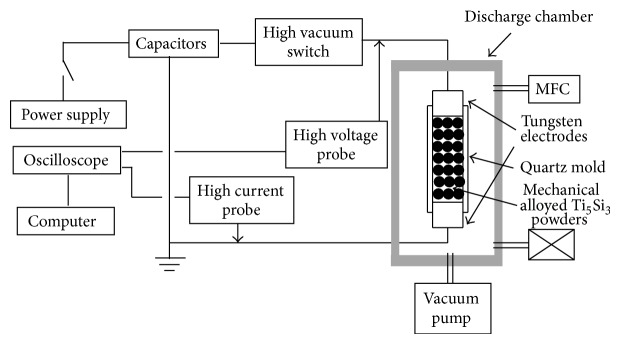
A schematic diagram of the experimental setup for the electrical discharge consolidation (EDC) technique.

0.34 grams of MAed powder was vibrated into a quartz tube with an inner diameter of 4.0 mm that had a tungsten electrode at the bottom and top. The discharging chamber was evacuated to 2 × 10^−2^ torr. A capacitor bank of 450 *μ*F was charged with two different electrical input energies (7.0 and 8.0 kJ). The charged capacitor bank instantaneously discharged through the MAed powder column without applying any pressure by on/off high vacuum switch which closes the discharge circuit. The voltage and current that the powder column experiences when the circuit is closed were simultaneously picked up by a high voltage probe and a high current probe, respectively. Outputs from these probes are fed into a high speed oscilloscope that stores them as a function of discharge time. The overall process is referred to as electrical discharge consolidation (EDC). A schematic of the EDC apparatus is shown in Figure 1.

The phase compositions of the MAed powder and EDC compacts were investigated by X-ray diffraction (XRD) using Cu K_*α*_ radiation. Each EDC compact was sliced every two millimeters and the resulting cross-sections were examined under scanning electron microscopy (SEM) and transmission electron microscopy (TEM). The average hardness values were obtained from at least 20 measurements on the cross-sections of each sample.

## 3. Results and Discussion

**Figure 2:**
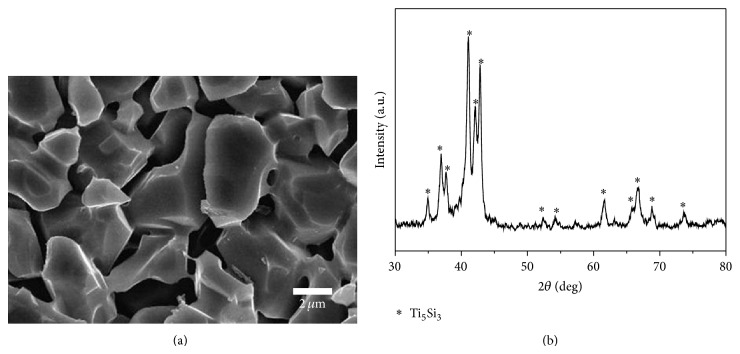
(a) SEM micrograph and (b) XRD patterns of MAed Ti-37.5 at.% Si powder mixture.

Figure 2(a) shows SEM micrograph of the MAed powder with a mean particle size of 3.4 *μ*m, which was used in current experiment. XRD patterns of the powder, shown in Figure 2(b), confirmed that the powder is mainly composed of Ti_5_Si_3_ phase.

**Figure 3:**
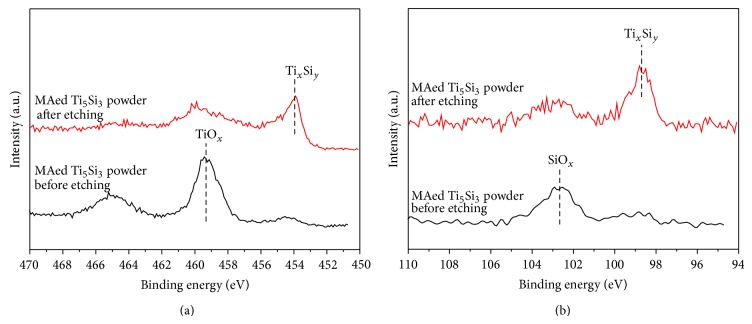
XPS narrow scan spectra of the (a) Ti 2p and (b) Si 2p region of MAed Ti_5_Si_3_ powder before and after light Ar^+^ etching for 5 minutes.

To investigate the surface chemical states of MAed Ti_5_Si_3_ powder, XPS was carried out.  Figure 3(a) shows narrow scan spectra of the Ti 2p region before and after light Ar^+^ etching for 5 minutes. For the MAed Ti_5_Si_3_ powder before etching, a Ti 2p_3/2_ peak at 459.2 eV is shown, with 5.8 eV splitting between the Ti 2p_1/2_ and Ti 2p_3/2_ peaks. The Ti 2p_3/2_ peak at 459.2 eV corresponds to TiO_*x*_, implying that the surface of MAed Ti_5_Si_3_ powder is primarily in the form of titanium oxide [16, 17]. However, after etching the MAed Ti_5_Si_3_ powder, the Ti 2p_3/2_ peak shifted to lower binding energy, 453.4 eV, which indicates the presence of titanium silicide [18]. It can thus be known that the MAed Ti_5_Si_3_ powder was lightly oxidized.  Figure 3(b) shows narrow scan spectra of the Si 2p region before and after light Ar^+^ etching for 5 minutes. For the MAed Ti_5_Si_3_ powder before etching, a Si 2p peak at 102.5 eV corresponds to SiO_*x*_ [18]. After etching the MAed Ti_5_Si_3_ powder, the Si 2p peak shifted to lower binding energy, 98.2 eV, which indicates the presence of titanium silicide [18]. This result also supports that the MAed Ti_5_Si_3_ powder was lightly oxidized.

**Figure 4:**
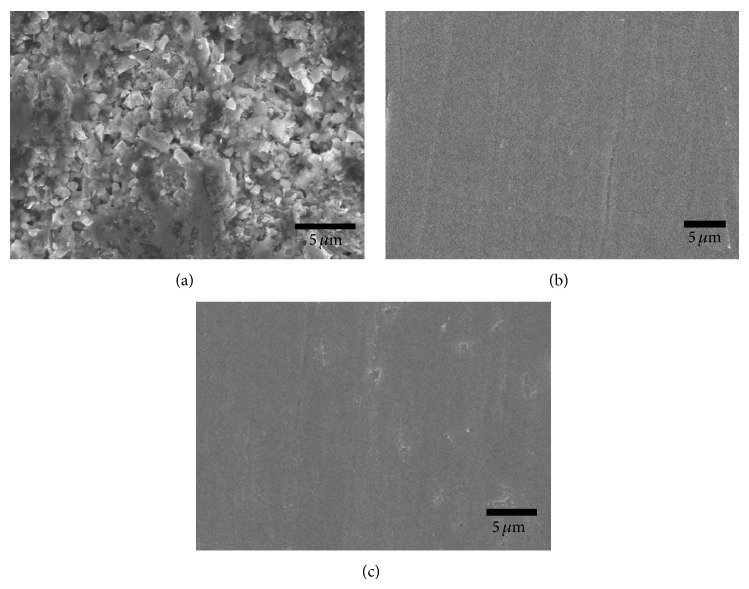
SEM micrographs of the cross-sections of consolidated Ti_5_Si_3_ compacts obtained by (a) hot-pressing at 1200°C in a vacuum of 2 × 10^−6^ torr for two hours with a pressure of 10 tons and electrical discharge consolidation using (b) 7.0 kJ and (c) 8.0 kJ of input energy.

**Figure 5:**
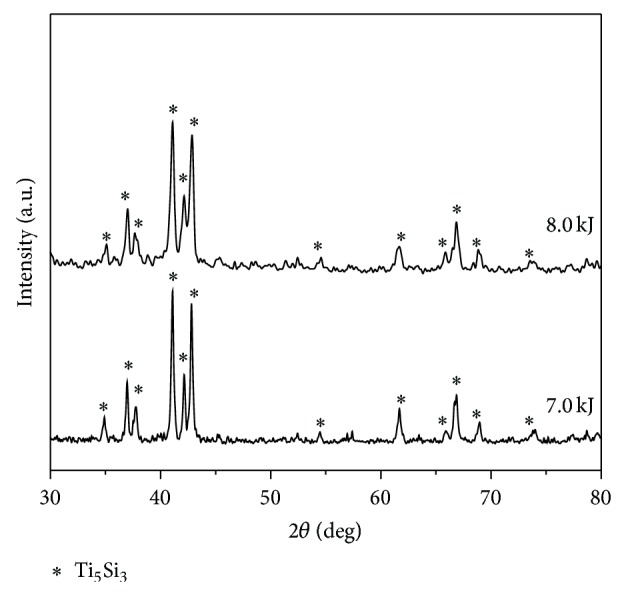
XRD patterns of the Ti_5_Si_3_ compacts obtained by electrical discharge consolidation of MAed Ti_5_Si_3_ powder using 7.0 and 8.0 kJ of input energy.

The MAed Ti_5_Si_3_ powder was consolidated by a conventional hot-pressing process. As shown in Figure 4(a), the consolidation process at 1200°C in a vacuum of 2 × 10^−6^ torr for two hours by applying a pressure of 10 tons did not successfully produce the compact in a bulk type, resulting in the formation of a porous structure. As listed in Table 1, the hardness of MAed Ti_5_Si_3_ powder was found to be about Hv 1120, but that of the hot-pressed Ti_5_Si_3_ compact decreased down to Hv 800. The decreased hardness can be attributed to the release of strain energy during a hot-pressing and also to the porous structure of the compact. The cross-section views of EDC Ti_5_Si_3_ compacts at the input energy of 7.0 and 8.0 kJ are shown in Figures 4(b) and 4(c), respectively. The compacts were composed of powder particles that were completely deformed and welded together by the electrical discharge. The density of the solid core of EDC Ti_5_Si_3_ compacts is approximately ~99% of theoretical value. From XRD patterns of the EDC Ti_5_Si_3_ compacts as shown in Figure 5, only peaks corresponding to the phase of Ti_5_Si_3_ have been found. It can be known that the unique phase of Ti_5_Si_3_ has not been altered by the electrical discharge process. The average crystallite size of EDC Ti_5_Si_3_ compacts was determined as 93–101 nm by using Suryanarayana and Grant Norton's formula [19]. Measured hardness of EDC Ti_5_Si_3_ compacts is also listed in Table 1, indicating that the hardness can be increased by the electrical discharge.

**Figure 6:**
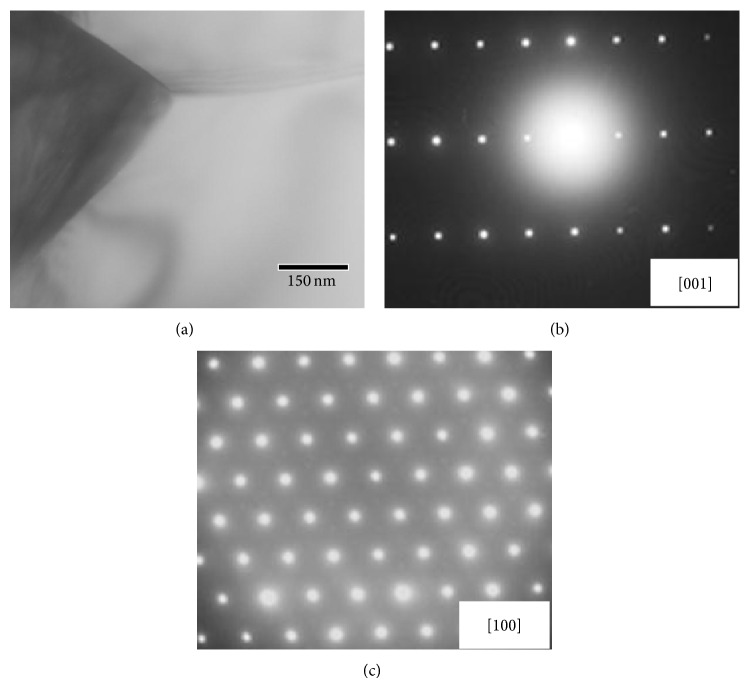
Typical TEM bright-field image (a) and selected area diffraction patterns ((b) and (c)) of the EDC Ti_5_Si_3_ compact at 7.0 kJ of input energy [13].

Figure 6 shows a typical TEM bright-field image (a) and selected area diffraction patterns ((b) and (c)) of the EDC Ti_5_Si_3_ compact discharged at 7.0 kJ of input energy [13]. TEM bright-field image in Figure 6(a) presents the facet grain boundary, which is quite flat suggesting that the grain boundaries of the Ti_5_Si_3_ compound are quite stable. The diffraction peaks in Figures 6(b) and 6(c) correspond to [001] and [100] zone axis of hexagonal Ti_5_Si_3_ compound (P6_3_/mcm), respectively. Based on the analysis of the diffraction patterns, a value of the lattice parameter for the EDC Ti_5_Si_3_ compact can be calculated as *a* = 7.42 Å and *c* = 5.17 Å, which is almost identical to the value of the lattice parameter in the standard hexagonal Ti_5_Si_3_ compound; that is, *a* = 7.46 Å and *c* = 5.15 Å [14]. This indicates that there is no compositional deviation even after the electrical discharge process. This result supports that physical breakdown of the oxide film of MAed powder occurs first in the initial stage of an electrical discharge.

**Figure 7:**
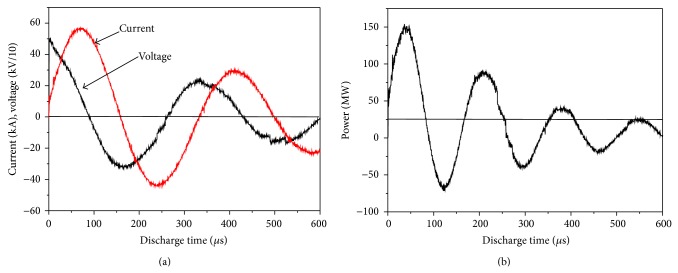
(a) Typical discharge curve measured current and voltage on oscilloscope and (b) typical power curve versus discharge time (discharge condition: 450 *μ*F, 7.0 kJ).

To investigate the consolidation mechanism of nanostructured Ti_5_Si_3_ solid compact by electrical discharge, electrical discharging characteristics were considered in terms of input energy and capacitance under current experimental conditions. A typical discharge curve (Figure 7(a)) shows voltage and current in terms of discharge time. 450 *μ*F of capacitance and 5.58 kV of input voltage were employed to yield 7.0 kJ. The input energy (*E*) is predetermined by controlling input voltage (V) according to

(1) $$\begin{array}{c} E=\frac{C{\text{V}}^{2}}{2}, \end{array}$$

where *C* is the capacitance of a capacitor. Figure 7(a) shows that the peak current was 58.4 kA and the peak voltage was 5.04 kV. From the results shown in Figure 7(a), the power (watt) curve is plotted in Figure 7(b) against the discharge time. The power was obtained from the following equation:

(2) $$\begin{array}{c} P\left(\text{watt}\right)=\text{current}\left(\text{A}\right)\times \text{voltage}\left(\text{V}\right)=I^{2}R\;\left(\text{J}/\sec\right). \end{array}$$

The discharge times for the duration of the first cycle at two different input energies are identical to be approximately 159 *μ*sec. The amount of heat generated (Δ*H*) during a discharge can be obtained by using

(3) $$\begin{array}{c} \Delta H=\sum \left[i^{2}\left(t\right)R\left(t\right)\Delta t\right]. \end{array}$$

Typical discharge characteristics under the current conditions are tabulated in Table 2 in terms of peak current, peak voltage, discharge time, and Δ*H*. It is known that Δ*H* increases with an increase in input energy at constant capacitance.

As a usual sintering process, the consolidation of metal powder requires a heat. To understand the effects of Δ*H* as one of discharge characteristics for the consolidation process, the temperature rise (Δ*T*), which is caused by an input energy, is now considered and estimated using

(4) $$\begin{array}{c} W=mCp\Delta T, \end{array}$$

where *m* is the mass of the MAed Ti_5_Si_3_ powder and *Cp* is the specific heat of Ti_5_Si_3_. The electrical input power (*W*) was calculated by integrating current and voltage as a function of discharge time. The resulting data for the heat generated by the electrical discharge process are listed in Table 3. It can be known that the electrical discharge produces the heat significantly greater than the melting temperature of Ti_5_Si_3_. Such a heat generated through the MAed Ti_5_Si_3_ powder is supposed to be high enough to vaporize the Ti_5_Si_3_ powder. However, the duration of the heat rise as 159 *μ*sec could be too short for the complete vaporization process, resulting in the phase transformation into liquid. Moreover, it can be expected that the consolidated Ti_5_Si_3_ compact contains some pores since the electrical discharge process of Ti_5_Si_3_ powder was carried out without applying any pressure. Therefore, one possible force which can pressurize the liquidus powder can be considered as a function of input energy.

**Figure 8:**
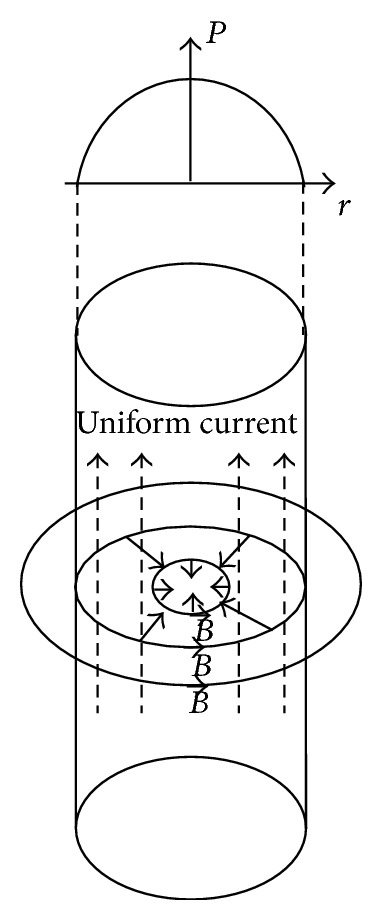
Linear pinch effect with a uniform current distribution.

When a capacitor bank is discharged through a powder column, a long cylindrical metal powder column conducting an axial current, distributed axisymmetrically as shown in Figure 8, tends to contract radially inwards. At this moment the magnetic field generated by the current flow causes a diametric contraction, which is known as the pinch effect [20]. The magnitude of the magnetic field (*B*) can be obtained by using

(5) $$\begin{array}{c} B=\frac{1}{2}\mu rj, \end{array}$$

where *μ* is the permeability, *j* is the current density, and *r* is the distance from the center of the powder column. The resulting pinch pressure (*P*) is the mechanical force acting on the powder column that will produce a solid core. The pinch pressure is given by

(6) $$\begin{array}{c} P=\frac{\mu j^{2}\left(a^{2}-r^{2}\right)}{4}, \end{array}$$

where *a* is the radius of the cylindrical powder column. Allow the diameter (2*a*) of the contact region to be approximately one-tenth of the diameter of an average powder particle, as is often the case in solid mechanics [21]. MAed Ti_5_Si_3_ powder particles are considered to be stacked in such a linear manner that only one contact point is formed, resulting in the parallel straight current passage. The geometrical parameters needed for estimating the pinch pressure can be obtained and are tabulated in Table 4. Using these parameters, the maximum pinch pressure can be estimated in the center of the contact area (at *r* = 0). The resulting pinch pressures calculated under current experimental conditions are also listed in Table 3. It can be known that the pinch pressures between 300 and 322 MPa by the discharge were generated and could pressurize the liquidus powder particles, producing a bulk-typed Ti_5_Si_3_ compact without containing pores.

**Figure 9:**
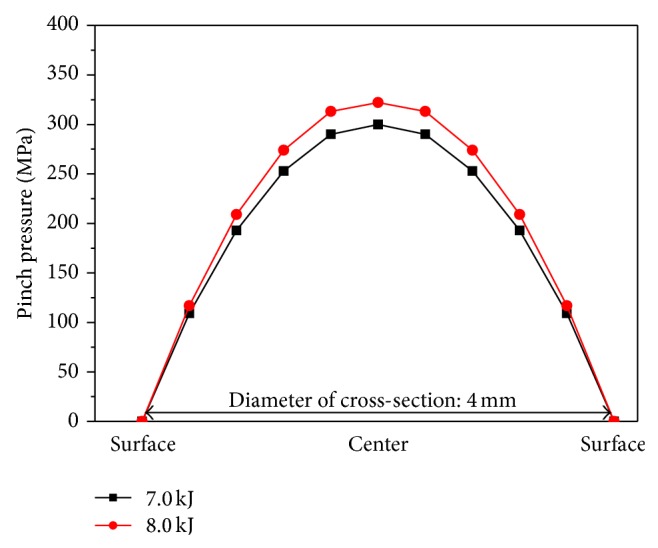
Distribution of pinch pressure generated on the cross-section of EDC Ti_5_Si_3_ compact.

Since the pinch pressure is maximal at the center of Ti_5_Si_3_ powder column, its distribution across the cross-section of the compact should be different. The distribution of pinch pressures generated across the Ti_5_Si_3_ powder column with two different input energies is shown in Figure 9. The pinch pressure decreases down to 0 at the surface of the Ti_5_Si_3_ powder column. Since the pinch pressure is maximal at the center of the powder column, the solid core is formed easily, especially in the center of the Ti_5_Si_3_ compact. Therefore, it is very logical that the heat generated is the required parameter to melt the MAed Ti_5_Si_3_ powder particles and the pinch pressure can condense them without allowing a formation of pores across the compact.

**Figure 10:**
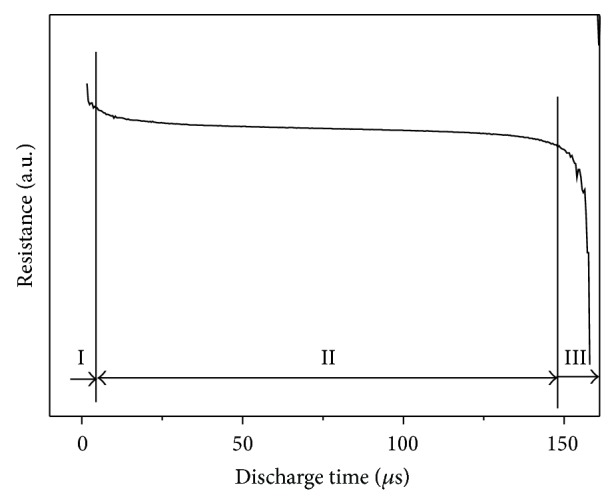
Resistance variation of MAed Ti_5_Si_3_ powder column calculated from the voltage and current recordings during an electrical discharge.

Figure 10 shows the resistance change through the MAed Ti_5_Si_3_ powder column during an electrical discharge, which was determined from the recordings of voltage and current. It can be seen that there are three distinct regions: 0 to 6 *μ*sec as stage 1, 7 to 146 *μ*sec as stage 2, and 147 to 159 *μ*sec as stage 3. In stage 1, electronic and physical breakdown of the oxide layer of MAed Ti_5_Si_3_ powder occurred, causing the rapid drop of resistance. In stage 2, the resistance decreased very slowly. The heat generated during a discharge would liquefy the MAed Ti_5_Si_3_ powder. Both condensation and densification of melted Ti_5_Si_3_ powder are promoted by the pinch force, especially in the center of the powder column. In stage 3, another rapid drop of resistance occurred. The rapid cooling occurs in this stage, resulting in the preservation of nanosized crystallite of Ti_5_Si_3_ compact.

**Figure 11:**
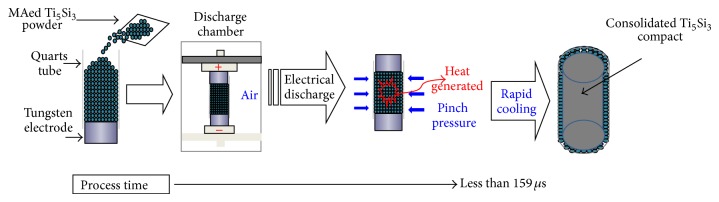
Schematic illustration for the formation of nanostructured Ti_5_Si_3_ compact by an electrical discharge of MAed Ti_5_Si_3_ powder.

Figure 11 shows the formation sequence of nanostructured Ti_5_Si_3_ compact by the electrical discharge of MAed Ti_5_Si_3_ powder, indicating that the heat generated, pinch pressure, and rapid cooling are required parameters for the consolidation process of nanostructured Ti_5_Si_3_.

## 4. Conclusions

Electrical discharges of mechanical alloyed Ti-37.5 at.% Si powder mixture using a capacitance of 450 *μ*F at input energies of 7.0 and 8.0 kJ were carried out without applying any external pressure. The MAed Ti_5_Si_3_ powder was successfully consolidated in times as short as 160 *μ*sec into a solid bulk of Ti_5_Si_3_ compact with nanosized crystallites. It is proposed that, during an electrical discharge, physical breakdown of the oxide film of MAed Ti_5_Si_3_ powder occurs first. Both melting and condensing the Ti_5_Si_3_ powder are promoted by the heat and the pinch force especially in the center of the powder column, respectively. And then rapid cooling occurred, resulting in the formation of nanostructured Ti_5_Si_3_ compact.

## Figures and Tables

**Table 1:** Microhardness of MAed Ti_5_Si_3_ powder, hot-pressed Ti_5_Si_3_ compact, and EDC Ti_5_Si_3_ compacts.

MAed Ti_5_Si_3_ powder	Hot-pressed Ti_5_Si_3_ compact	EDC Ti_5_Si_3_ compact	
		Input energy	
		7.0 kJ	8.0 kJ
Hv 1120	Hv 800	Hv 1410	Hv 1450

**Table 2:** Peak voltage, peak current, discharge time, and heat generated (Δ*H*) during a discharge.

Capacitance (*μ*F)	Input energy (kJ)	Peak voltage (kV)	Peak current (kA)	Discharge time (*μ*F)	Δ*H* (J)
450	7.0	5.04	58.4	159	5880
450	8.0	5.36	60.8	159	6640

**Table 3:** Temperature rise (Δ*T*), current density (*j*), and pinch pressure (*P*) produced by an electrical discharge.

Input energy (kJ)	Temperature rise (°C)	Current density (A/m^2^)	Pinch pressure (MPa)
7.0	29,163	7.75 × 10^11^	300
8.0	33,371	8.06 × 10^11^	322

**Table 4:** Geometric parameters in the pinch pressure calculation.

Number of particles on cross-section of powder column	614
Contact area of particle (m^2^)	1.23 × 10^−10^
Mean cross-sectional area of powder particle (m^2^)	7.55 × 10^−8^
Radius of the powder column (m)	1.2 × 10^−4^
